# Effect of coconut and sesame oils on gingivitis

**DOI:** 10.6026/973206300200368

**Published:** 2024-04-30

**Authors:** Priyanka Vinod Bansal, Chinmayee Kulkarni, Vishnu Teja Obulareddy, Sravana Laxmi Penumaka, Kiran Kulkarni

**Affiliations:** 1 Prosthodontics and Oral Implantology, Wakad, India; 2 Rajashree Chatrapati Shahu Maharaj Govt. Medical college Kolhapur, India; 3 Virginia State Dental Association, Richmond, Virginia USA; 4 Department of Conservative Dentistry & Endodontist, Government Dental College & Hospital, Vijayawada, India

**Keywords:** Oil pulling, coconut oil, sesame oil, gingivitis

## Abstract

The effectiveness of Oil Pulling Therapy (OPT) with coconut (CO) and sesame oil (SO) on gingivitis patients is of interest. Forty patients were randomly distributed into group A and B for CO and SO respectively. Participants of group A were explained in detail about the OPT with CO and group B with SO along with their routine oral hygiene practice for 30 days. The mean plaque index of CO and SO reduced from 1.5 to 1.32 and 1.65 to 1.36 (p<0.05) respectively after 30 days. The mean gingival index of CO and SO declined from 1.12 to 0.9 and 1.1 to 0.81 respectively after 30 days (p<0.05) compared to initial scores. The mean no. of colonies in the case of CO and SO declined from 35.8 x 103 to 32.4 x 103 and 6.8 x 103 to 34.6 x 103 after 30 days (p<0.05). OPT reduced plaque and gingivitis, according to the results of one month. Hence, we must increase awareness about oil pulling, as this home therapy can prevent gingival diseases in countries with limited resources like ours.

## Background:

Gingivitis, an inflammation of the gingival tissues affects only the connective tissue and gingival epithelium. Based on the aetiology, severity, time span, and clinical features, there are several types of gingivitis [1]. A shiny surface, swelling, redness, bleeding on probing and pain are frequently seen clinical features of gingivitis [2]. Well-developed human dental plaque contains more than 300 different types of micro-organisms; some of these microorganisms have been studied on, while others remain unidentified. Currently, there is a general consensus among experts that at least 7-8 different species, including *A. actimomycetemcomitans*, *P. gingivalis*, *T. forsythia*, *P. intermedia*, *C. rectus*, and *Spirochetes*, are responsible for different forms of periodontal disease [3]. The most common type of gingivitis has been determined to be the chronic type produced by plaque. The maintenance of oral hygiene in patients can present challenges due to factors such as increased plaque retention caused by the time-consuming nature of mechanical plaque removal methods, lack of motivation and manual dexterity, incorrect brushing techniques, and insufficient utilisation of other oral hygiene aids such as floss, mouthwashes, and tongue cleaners [4]. For the best dental health maintenance, additional chemical plaque management techniques such mouthwashes are needed, but their prolonged usage is associated with certain drawbacks. Thus, a need for herbal products was recognised [5]. Oil pulling therapy (OPT) has been widely employed for many years as part of alternative medicine to prevent tooth decay, bleeding gums, oral malodor, throat dryness, and cracked lips, as well as to strengthen teeth, gums, jaws etc. OPT, also known as oil swishing, is a traditional Indian ayurvedic treatment involving the swishing of oil in the mouth for oral and systemic health benefits [6]. Even though refined oil also "pulls" bacteria, viruses, and protozoa from the buccal cavity, cold-pressed organic oils such as sesame oil, sunflower oil, and coconut oil are particularly beneficial. Since cold pressed oils do not contain Trans fats compared to commercial oils extracted with heavy petroleum based solvents, cold pressed oils are recommended for OPT [7]. Coconut oil (CO) and sesame oil (SO) have historically been recognised as the preferred oils for the practise of OPT [8]. Recent research has demonstrated that sesame oil and sunflower oil can effectively mitigate the development of plaque-induced gingivitis [9]. The root of Sesame (*Sesamum indicum*) is known to contain chlorosesamone, a compound that exhibits antifungal properties. Moreover, the presence of polyunsaturated fatty acids in SO serves to mitigate the occurrence of frees radical damage within the oral cavity [10]. However, there is no data on the comparative effects of CO and SO on the periodontium. Therefore, it is of interest to evaluate and compare the effectiveness of OPT with coconut and sesame oil on gingivitis patients.

## Materials and Methods:

## Study Sample:

The current study was carried out to evaluate and compare the effectiveness of OPT with coconut and sesame oil on gingivitis patients. Forty patients who were referred to the Department of Periodontology for gingivitis were selected and randomly distributed into group A and B for CO and SO respectively based on inclusion and exclusion criteria. The study duration was one year and institutional ethical approval was taken. The included patients were explained about the study and an informed consent was taken. All included patients had at least 20 permanent natural teeth having mild to moderate plaque induced gingivitis was selected. They were excluded if they had periodontal pockets or suppuration. They were also excluded if they were not systemically healthy or had used any antibiotics in the last 3 months or had any allergy to the oil used in the study.

## Study parameters:

The plaque index (PI) and gingival index (GI) of all the participants were used to record the severity of gingivitis and the thickness of plaque in the gingival area of the tooth on the indexed teeth. To record colony forming units (CFU) at baseline, all the subjects were instructed to gargle with normal saline (0.85% NaCl). This saline was collected in a sterile container and was serially diluted and inoculated in blood agar, MacConkey agar, and nutrient agar and were incubated at 37°C for 24 hours. After this incubation period, the number of colonies present in 1 ml of the saline is calculated [11].

## Total colony count was calculated by the following formula:

Number of bacteria / ml = Number of colonies dilution x Amount plated

## Study procedure:

All patients underwent scaling and root planing procedure after their baseline parameters were recorded. Participants of group A were explained in detail about the OPT with CO and group B with SO along with their routine oral hygiene practice for 30 days. 40 sachets of 10ml CO and SO were given to all 40 patients according to their groups. The patients had to gargle on an empty stomach in the morning after they brush their teeth for a period of 15 to 20 minutes until the oil turns viscous and slowly becomes thin and milky in consistency. The patients were called for follow up and all parameters were recorded again after 30 days.

## Statistical analysis:

The data was entered and analyzed using the Statistical Package for Social Sciences (SPSS) for Windows, Version 28.0. (Armonk, NY: IBM Corp) Confidence intervals were set at 95%, and a p-value ≤ of 0.05 was considered statistically significant. Wilcoxon signed rank test is used to compare plaque scores, gingival scores and CFU count before and after OPT

## Results:

The present study included a total of 40 patients. Mean plaque index of CO and SO reduced from 1.5 to 1.32 and 1.65 to 1.36 (p>0.05) respectively after 30 days compared to initial scores. However, no significant reduction was seen between both groups. The mean gingival index of CO and SO declined from 1.12 to 0.9 and 1.1 to 0.81 respectively after 30 days (p>0.05) compared to initial scores. However, no significant reduction was seen between the two groups. The mean no. of colonies in the case of CO and SO declined from 35.8 x 103 to 32.4 x 103 and 36.8 x 103 to 34.6 x 103 after 30 days (p>0.05). However, on comparison no significant reduction was seen in both cases.

## Discussion:

Many chemical plaque control methods have been proposed to prevent and treat gingivitis and prevent it from leading to periodontitis. However, supplementary oral hygiene measures, such as the use of chemical mouthwashes, can cause allergic reactions and long-term use results in loss of taste sensation and tooth discoloration [12]. Such disadvantages have led researchers to turn to more natural remedies. OPT is an old ayurvedic method of oral hygiene. There is some evidence that OPT can assist saliva in the elimination of harmful heavy metals. During OPT, salivary enzymes remove chemical, bacterial, and environmental toxins from the blood and expel them through the tongue [13]. According to Ayurveda, the tongue is linked to the heart, kidneys, small intestine, spine, lungs, etc thereby benefitting the whole body. When compared to gold standard mouthwashes, several studies have been documented where OPT proved to be equally beneficial in curbing plaque induced periodontal disease at least for a limited duration [14, 15, 16, 17, 18, 19]. Thus, 10ml of cold pressed oil sachets were handed out to the participants of both groups. The current study included a total 40 patients with no attrition for 30 days. Our study found that the PI and GI scores were reduced post OPT in both groups. The CFUs also reduced significantly for both groups. However, even though the oils individually performed well in their respective groups, the comparative evaluation revealed no statistically significant differences. Our study is in agreement with several studies. Shetty *et al* in a recent study of 2022 compared the effectiveness of OPT using CO and SO on patients with plaque-induced gingivitis. They showed that the severity of gingivitis had remarkably decreased in both groups on after one and two weeks. Both oils were found to be equally effective [20]. Another similarly conducted study by Kaliamoorthy *et al* in 2018 found significant reduction in the severity of gingivitis with CO and SO within one, two and three weeks. However, more significant decrease was found in CO group [21]. Our study also showed better results with CO although not statistically significant. With respect to microbiological assessment, several studies are in agreement. Sezgin *et al*., in 2023 compared the plaque inhibiting effects of OPT with those of SO or CO using a 4-day plaque regrowth study model to see which one was more effective. They discovered that either CO or SO produced outcomes that were comparable in terms of inhibiting the renewal of plaque and tooth discoloration [22]. In a study that was conducted by Pavithran *et al.* effectiveness of OPT with CO on S. Mutans count was evaluated. After oil pulling using pure CO, a significant reduction in number of CFUs of S. mutans was noted. However, there was no difference that could be considered statistically significant between SO and CO [23]. According to the findings of another study carried out by Saravanan and colleagues, oil pulling led to a significant decrease in gingival, plaque scores, and number of bacteria found in the mouth [24]. After one month and 10 days of utilising SO for OPT, Anand and colleagues reported a 20% reduction in the number of pathogens in their study. In addition to this, they found that dental caries severity had decreased. They discovered SO to possess a significant amount of antibacterial action against both *S.mutans* and *L.acidophilus*. They claimed toxins and bacteria from the body would potentially be evacuated via tongue, where they would then become coated up in oil before being expelled from the body [11]. In an another invitro research using an oral biofilm model, SO had antibacterial action against *S.mutans* and CO had antibacterial activity against both C. albicans and *S. mutans* was observed [26]. After performing gum massage with SO, olive oil, and CO on 32 individuals, Singla *et al.* noticed a significant decrease in the mean number of *S.mutans* and *Lactobacilus* from the patient saliva, as well as a decrease in the subject’s plaque and gingival indices. After brushing their teeth, the participants in the study used their index finger to apply 2 millilitres of oil to their gums and massage them in a circular motion for ten minutes thereafter for three weeks. The subjects in this study did not report any unfavourable impacts [27]. Dani N *et al.*, evaluated antiplaque effects of OPT with SO. After scaling & root planing, 20 participants were assigned at random to conduct an OPT for a period of 2 weeks, while remaining 20 subjects were administered chlorhexidine mouth wash for the same period of time. Following 14 days of OPT, the plaque, gingival indices, and CFUs of aerobic bacteria were decreased for patients using SO. They discovered that using SO to treat plaque-induced gingivitis was as effective as using chlorhexidine [28]. The superior properties of CO are owed to lauric acid that has anti-inflammatory and antimicrobial properties. It is possible for it to combine with the salivary alkalis like sodium hydroxide and bicarbonates, for producing a compound that is similar to sodium laureate soap like substance, that has the ability to minimise plaque adhesion and accumulation, as well as cleaning actions [29]. CO also has the presence of monolaurin which is effective against pathogens such as *S. Aureus*, *Enterobacter spp*, *H. Pylori*, *Candida spp*., *E Vulneris* etc. Monolaurin is theorised to cause bacterial death by modifying the bacterial cell wall, and permeating the cell membrane, causing it to become disrupted, as well as by blocking enzymes that are involved in the creation of energy and the transfer of nutrients [30]. The antioxidant, antibacterial, and detoxifying properties of sesame oil come from sesamin, sesamolin, and sesaminol, which are all found in sesame oil [7]. Additionally, it inhibits the oxidation of lipids. Additionally, the price of SO is more than five times lower than that of chlorhexidine. Since it has such a wide range of applications, sesame oil has earned the reputation of being the most valuable of all oil seed crops [31]. OPT produces antioxidants that destroy microorganisms by damaging their cell walls [31]. These oils work on the lipid layer of bacterial cell membranes and adhere to the oil. During OPT, the oil becomes emulsified, expanding its surface area. Oil emulsification commences after five minutes of OPT [32]. This oil forms a coating on the tooth surface and gums, thereby inhibiting bacterial co-aggregation and plaque formation [29]. The oral cavity is thus cleansed of the plaque-forming bacteria that could lead to various dental ailments like dental caries, gingivitis, periodontitis, and poor breath. The gums not only regain their color and look healthier, but also a reduction of gingival bleeding is seen. OPT prevents gingivitis, dental caries, periodontitis and oral candidiasis, reduces tooth pain, and mobility of teeth, thus achieving optimal oral hygiene [33]. The study has a few limitations. Firstly, the sample size could be larger. Secondly, a longer follow up period is required to fully understand plaque re-accumulation and various effects of individual oils. Thirdly, patient preference could also be noted as the oils have different viscosity. CO is thicker than SO and an individual needs to swish it for at least 5 to 10 minutes. It could be a tedious process for some as mouthwashes are thinner and require a lesser time of swishing. Due to various and diverse benefits, oil pulling must be recommended in patient counselling as an additional aid as oils are proven to be beneficial and available readily in the comfort of their homes and the process is cost effective.

## Conclusion:

Untreated gingivitis can lead to periodontal disease. Effective prevention that inhibits disease progression and maintains tissue health is crucial. Oil Pulling reduces plaque and gingivitis, according to the results. It is necessary to investigate a home remedy, such as oil pulling that saves time and money while improving overall health. Therefore, we must increase awareness about oil pulling; this overlooked home therapy that can prevent disease in countries with limited resources like ours.

## Funding:

None

## Figures and Tables

**Table 1:** Clinical and microbiological findings

**Group**	**CO**			**SO**		
Parameters	Baseline	30 days follow up	p-value	Baseline	30 days follow up	p-value
Plaque index	1.5±0.14	1.32±0.29	0.31	1.65±0.6	1.46±0.31	0.41
Gingival index	1.12±0.7	0.9±0.6	0.24	1.1±0.73	0.81±0.52	0.34
Colony forming units	35.8 x 103	32.4 x 103		36.8 x 103	34.6 x 103	
